# Fluid balance trajectories and prognosis in patients with acute myocardial infarction complicated by cardiogenic shock: a group-based trajectory model approach

**DOI:** 10.3389/fcvm.2025.1674197

**Published:** 2026-01-05

**Authors:** Chunmei Zhang, Zhiyi Xie, Guangyu Lin, Qitian Zhang

**Affiliations:** Department of Cardiology, Zhangzhou Affiliated Hospital of Fujian Medical University, Zhangzhou, Fujian, China

**Keywords:** acute myocardial infarction, cardiogenic shock, GBTM, fluid balance, MIMIC database, prognosis

## Abstract

**Background:**

Fluid management is crucial in the treatment of patients with acute myocardial infarction complicated by cardiogenic shock (AMI-CS), yet the optimal strategy remains unclear. This study aims to evaluate the association between fluid balance (FB) trajectories and prognosis in AMI-CS patients.

**Methods:**

This study utilized data from the MIMIC-IV database, including patients diagnosed with AMI-CS. A Group-Based Trajectory Model (GBTM) was applied to identify patient groups with similar FB trends. The association between different FB trajectories and patient survival was assessed using Kaplan–Meier survival analysis and Cox regression models. Additionally, subgroup and sensitivity analyses were conducted to validate the robustness of the results.

**Results:**

A total of 533 AMI-CS patients were included. The 4-group trajectory model showed good fit (AIC = 19,937.75; minimum AvePP = 0.81). Four FB trajectory patterns were identified: trajectory 1 (stable negative balance), trajectory 2 (rapid decline to negative balance), trajectory 3 (persistent positive balance), and trajectory 4 (high-level decreasing). Kaplan–Meier survival analysis revealed that patients in trajectories 1 and 2 had higher survival rates, while those in the fluid overload group had a significantly higher risk of death compared to the non-overload group. Cox regression analysis further demonstrated that, compared to trajectory 2, trajectory 3 was associated with a significantly increased mortality risk, while trajectory 1 showed no statistically significant difference. Subgroup and sensitivity analyses were consistent, confirming the robustness of the study findings.

**Conclusion:**

Among the dynamic FB patterns in AMI-CS patients, stable negative balance or rapid transition to negative balance is associated with the best prognosis. The GBTM approach helps identify different risk strata within the AMI-CS patient population.

## 1 Introduction

Cardiogenic shock (CS) is one of the most severe complications of acute myocardial infarction (AMI), mainly resulting from severe cardiac dysfunction that leads to inadequate perfusion of vital organs and subsequent multi-organ failure (1). The incidence of CS among AMI patients is approximately 5%–10% (2, 3). Currently, acute myocardial infarction complicated by cardiogenic shock (AMI-CS) remains a clinical syndrome with an extremely high fatality (4), with a 30-day mortality approaching 40% and a one-year mortality nearing 50% (5). Although timely reperfusion can improve patient prognosis to some extent, the overall mortality rate of AMI-CS patients has not significantly decreased over the past decades (4). A deeper investigation into the key factors affecting the prognosis of AMI-CS patients is of great clinical significance for optimizing treatment strategies.

Fluid management plays a crucial role in the treatment of AMI-CS patients, serving as a key intervention to maintain hemodynamic stability, increase cardiac output, and improve tissue perfusion (6, 7). However, AMI-CS patients often experience complex pathophysiological changes such as volume depletion and fluid redistribution (8). Fluid balance (FB) represents the net difference between fluid input and output, reflecting the overall hydration status of critically ill patients. However, accurately assessing FB remains challenging, as traditional methods such as intake–output records or body weight changes cannot fully capture fluid distribution. Although new techniques such as bioelectrical impedance analysis and ultrasound-based assessment have been proposed to improve FB evaluation, their clinical implementation remains difficult (9, 10). Although fluid therapy may be required to optimize preload and maintain perfusion, its use should be guided by the patient's volume status, as inappropriate administration can lead to fluid overload (FO), resulting in microcirculatory dysfunction and potential harm (11). In recent years, several studies have focused on the potential impact of fluid balance (FB) on the prognosis of AMI-CS. Some studies have shown that positive FB is significantly associated with higher mortality and severe acute kidney injury (AKI), suggesting that fluid imbalance may be an important adverse prognostic factor (12–14). However, other research has found that, for patients with severe heart failure and/or CS, ICU (intensive care unit) discharge FO appears to be unrelated to 30-day mortality (15). Differences in the definition of FO, assessment time windows, and outcome measures across studies have limited the consistency and comparability of results. Additionally, the fluid management in these studies was based on FB status at specific time points and did not reflect the dynamic changes in overall FB. Therefore, the impact of fluid management on the prognosis of AMI-CS patients still needs to be clarified through further high-quality studies.

Group-Based Trajectory Modeling (GBTM) can more accurately describe and understand heterogeneity and similarity between individuals (16) and is used to explore dynamic changes in individuals (16). We aim to explore the association between FB changes and prognosis in AMI-CS patients in the ICU. This study intends to use the GBTM approach to analyze FB trajectories in AMI-CS patients and reveal the association between different trajectory patterns and 30-day survival. The findings of this study will help optimize fluid management strategies for AMI-CS patients, formulate personalized treatment plans based on high-risk subtypes, thereby improving treatment outcomes, saving medical resources, and shortening the clinical uncertainty period.

## 2 Materials and methods

### 2.1 Data source

The data for this study were sourced from the MIMIC-IV (version 3.1) database (17). This publicly available de-identified intensive care unit (ICU) database includes comprehensive health records of patients admitted to the ICU at the Beth Israel Deaconess Medical Center in the United States between 2008 and 2022. Since all data have been de-identified, this study does not require written informed consent from patients nor approval from a research ethics committee or institutional review board. The corresponding author, Qitian Zhang, has completed the CITI certification and signed the PhysioNet Data Use Agreement (DUA), thereby obtaining authorized access to the MIMIC-IV database. The study strictly adheres to the guidelines of the STROBE statement for reporting epidemiological observational studies (18).

### 2.2 Study population

We selected patients with AMI-CS. AMI was defined by ICD-9 code prefix 410 or ICD-10 prefix I21, while CS was defined by ICD-9 code 785.51 or ICD-10 code R57.0. The inclusion criteria were: (1) age greater than 18 years and less than 100 years; (2) first ICU admission; (3) ICU stay ≥3 days to ensure sufficient data for dynamic fluid balance trajectory analysis. The exclusion criteria were: (1) fewer than 3 measurements of FB within 7 days; (2) missing more than 10% of variables; (3) exclusion of patients with missing body weight data for FB calculation; (4) patients who died within 72 h of ICU admission, as their short survival time did not allow adequate evaluation of FB trajectories.

### 2.3 Variables and outcomes

We extracted data on FB and prognosis indicators for AMI-CS patients during their first 1–7 days in the ICU, as well as the following variables: (1) Demographics: age, gender, body weight; (2) Vital Signs: temperature, heart rate, respiratory rate, blood pressure; (3) Laboratory Tests: complete blood count, blood biochemistry, coagulation function, blood gas analysis, myocardial enzymes, etc.; (4) Comorbidities: atrial fibrillation, hypertension, diabetes, hyperlipidemia, chronic obstructive pulmonary disease, pneumonia, chronic kidney disease; (5) Medications: ACEI/ARB, β-blockers, diuretics, vasopressor agents; (6) Other: use of CRRT, mechanical ventilation, presence of sepsis, presence of acute kidney injury, and severity scores (SOFA, Charlson score). Data extraction was performed using Navicat Premium 17.0 software and Structured Query Language (SQL) from the MIMIC-IV database, recording continuous fluid input and output data from day 1 to day 7. Laboratory indicators were extracted as the average value within the first 24 h of ICU admission.

FB was calculated using the following formula: FB = (Total Fluid Input - Total Fluid Output)/Initial Body Weight (kg). FO was calculated using the formula: (Cumulative FB/Initial Body Weight) × 100%. FO was defined as a FB exceeding 10% of the initial body weight (19). We summarized and calculated the daily fluid balance based on the data from the inputevents and outputevents tables in the MIMIC-IV database, using timestamps to match fluid input and output records. The primary outcome was the 30-day in-hospital mortality, defined as the patient's survival status within 30 days of ICU admission.

### 2.4 Group-Based trajectory modeling

This study used Group-Based Trajectory Modeling (GBTM) to model the dynamic changes in FB. GBTM is a semi-parametric method for analyzing longitudinal data and is used to identify subgroups with similar developmental trajectories (20). A cubic polynomial was used to fit the FB data, and the optimal number of trajectory groups was determined by stepwise comparison of different group models. The model's quality was evaluated based on the following criteria: (1) Bayesian Information Criterion (BIC) and Akaike Information Criterion (AIC), with smaller values indicating better model fit; (2) The average posterior probability (AvePP) should be greater than 0.7, indicating high accuracy of individual subgroup classification; (3) The sample proportion of each trajectory group should not be less than 5%; (4) The Odds of Correct Classification (OCC) minimum value should be greater than 5.0, with larger values indicating better model fit; (5) Based on statistical fit, a comprehensive judgment was made combining the model's simplicity and clinical interpretability to determine the best model.

### 2.5 Statistical analysis

All statistical analyses were performed using R software [version 4.4.3, (http://www.r-project.org)]. The normality of continuous variables was assessed using the Shapiro–Wilk test. Normally distributed data were presented as mean ± standard deviation, with group comparisons made using one-way analysis of variance (ANOVA). Non-normally distributed data were presented as median (interquartile range) and analyzed using the Kruskal–Wallis test. Categorical variables were presented as frequencies and percentages, with group comparisons made using Chi-square (*χ*^2^) test or Fisher's exact test. For missing data, variables with missing data exceeding 10% were excluded, while other missing values were imputed using the K-Nearest Neighbors (KNN) method. Survival analysis was conducted using the Kaplan–Meier curve, and the Log-rank test was used to compare survival differences among different FB trajectory groups. To evaluate multicollinearity among covariates, a correlation heatmap was generated and the Variance Inflation Factor (VIF) was calculated. Variables with a correlation coefficient greater than 0.5 or a VIF exceeding 5 were selected. A univariate and multivariate Cox proportional hazards model was used to analyze the association between FB trajectories and 30-day mortality, with results presented as Hazard Ratios (HR) and 95% Confidence Intervals (CI). Four Cox regression models were constructed: (1) unadjusted model; (2) adjusted for age and gender; (3) further adjusted for laboratory indicators (sodium, anion gap); (4) further adjusted for medication use (ACEI/ARB and *β*-blockers). Subgroup analyses were conducted by age (≤65 years vs. >65 years), gender (male vs. female), and SOFA score (<6 vs. ≥6) to assess the stability of associations across groups. To further validate the robustness of the results, sensitivity analyses were performed by excluding patients with hypotension (systolic blood pressure <90 mmHg) or chronic kidney disease (CKD). All statistical tests were two-sided, with a *P* value < 0.05 considered statistically significant.

## 3 Results

### 3.1 Baseline characteristics

**Figure 1:**
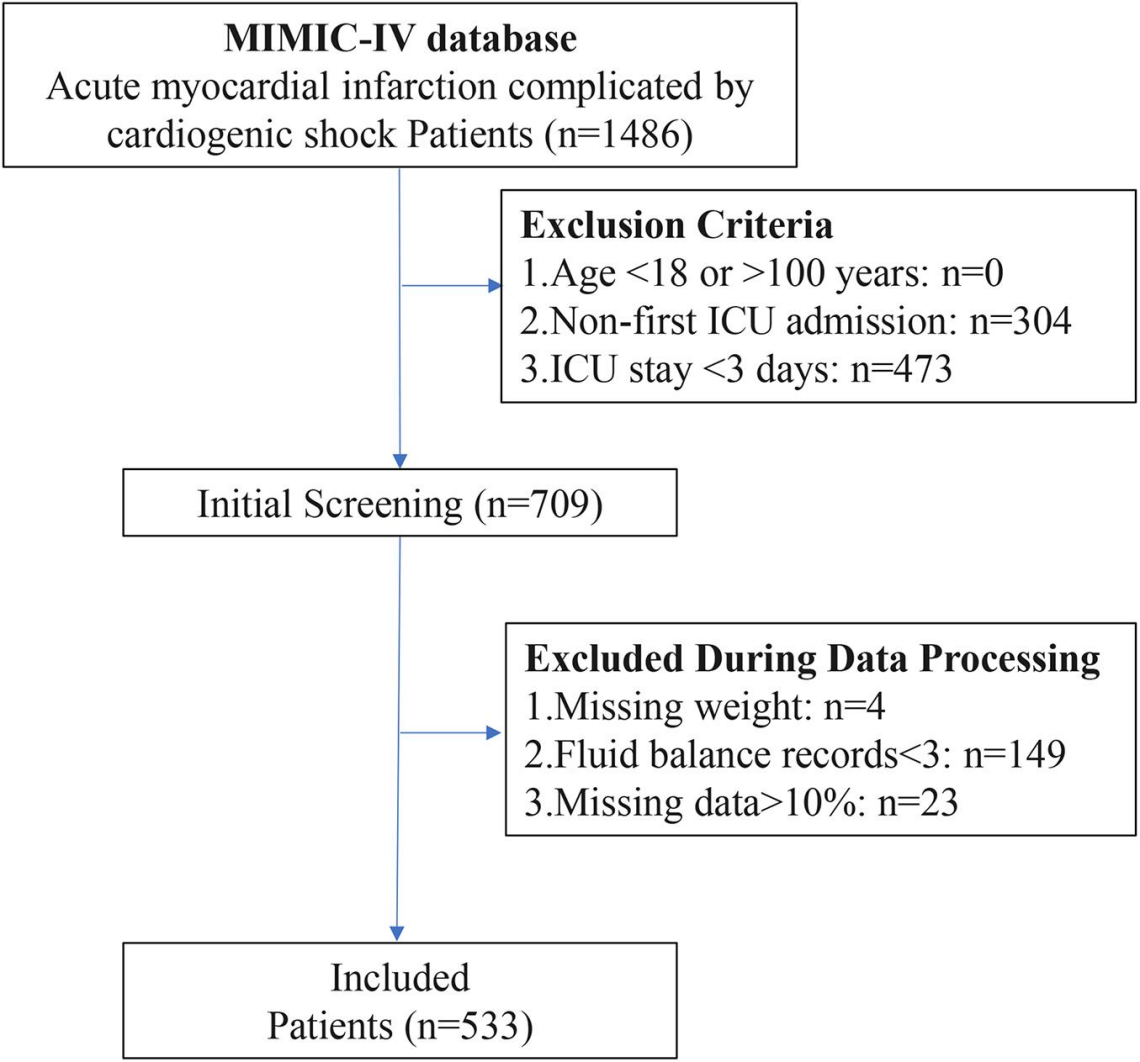
Flowchart of AMI-CS patient selection process.

A total of 1,486 AMI-CS patients were screened from the MIMIC-IV database. After applying the exclusion criteria, 533 patients were ultimately included in the study cohort (Figure 1). The variable screening is shown below, including those with a high proportion of missing data (Supplementary Figure S1), large correlations (Supplementary Figure S2), and strong multicollinearity (Supplementary Figure S3), which were excluded. Table 1 displays the baseline characteristics of the patients. The median age of all AMI-CS patients was 73.0 years, with an average weight of 79.0 kg. Of the patients, 336 (63.0%) were male, and 190 (35.6%) had chronic kidney disease (CKD). The average SOFA score at ICU admission was 8.0, and the Charlson score was 7.0.

**Table 1:** Baseline characteristics of patients with acute myocardial infarction complicated by cardiogenic shock in different fluid balance trajectory groups.

Variables	Total (*n* = 533)	1 (*n* = 307)	2 (*n* = 74)	3 (*n* = 96)	4 (*n* = 56)	*P*
Demographics						
Age (years)	73.0 [63.0;82.0]	73.0 [64.0;82.0]	71.0 [62.0;80.8]	73.0 [63.8;81.0]	73.0 [63.8;81.0]	0.672
Gender						0.028
Female	197 (37.0%)	112 (36.5%)	20 (27.0%)	34 (35.4%)	31 (55.4%)	
Male	336 (63.0%)	195 (63.5%)	54 (73.0%)	62 (64.6%)	25 (44.6%)	
Weight (kg)	79.0 [67.6;90.0]	78.3 [69.0;90.0]	83.0 [73.7;92.8]	80.4 [70.0;93.1]	67.9 [59.0;84.6]	0.001
Vital signs						
T (℃)	36.7 [36.4;37.0]	36.7 [36.4;36.9]	36.7 [36.4;37.1]	36.7 [36.5;37.0]	36.7 [36.5;37.0]	0.68
HR (bpm)	88.0 [77.0;103.0]	87.0 [74.0;101.0]	87.0 [80.0;109.5]	89.5 [77.0;106.0]	89.5 [82.8;110.0]	0.097
RR (bpm)	20.0 [16.0;24.0]	20.0 [16.0;24.0]	17.0 [14.0;23.0]	20.0 [17.0;24.0]	21.0 [17.0;26.0]	0.012
SBP (mmHg)	109.0 [95.0;125.0]	113.0 [98.5;127.0]	104.5 [94.0;120.8]	108.0 [93.8;122.0]	106.5 [89.8;121.2]	0.063
SpO2 (%)	97.0 [94.0;100.0]	97.0 [94.0;99.0]	97.0 [94.0;100.0]	98.0 [95.0;100.0]	98.0 [95.0;100.0]	0.107
Laboratory indicators						
WBC (m/uL)	13.6 [10.3;18.5]	12.8 [9.9;17.9]	14.6 [11.2;19.6]	15.4 [11.3;18.5]	15.2 [9.3;21.5]	0.099
PLT (K/uL)	212.5 [153.0;275.0]	213.0 [159.0;273.5]	211.2 [150.2;297.5]	214.5 [154.2;278.0]	188.5 [139.8;260.5]	0.553
Hb (g/dL)	10.8 [9.3;12.8]	11.1 [9.5;13.3]	10.5 [8.8;12.4]	10.6 [9.3;12.0]	10.8 [9.2;12.0]	0.036
RDW (%)	14.5 [13.4;15.9]	14.5 [13.5;15.9]	14.1 [13.1;15.1]	14.6 [13.7;16.0]	14.1 [13.3;16.3]	0.045
Na (mEq/L)	138.0 [135.0;141.0]	137.0 [134.0;140.0]	138.0 [136.0;140.8]	138.0 [135.0;141.0]	140.0 [136.0;142.0]	0.026
K (mEq/L)	4.3 [3.9;4.9]	4.3 [3.9;4.8]	4.2 [4.0;4.7]	4.5 [4.0;5.0]	4.2 [3.9;5.0]	0.163
Ca (mg/dL)	8.3 [7.8;8.7]	8.4 [8.0;8.8]	8.1 [7.3;8.5]	8.4 [7.7;8.8]	8.0 [7.3;8.3]	<0.001
GLU (mg/dL)	172.0 [126.0;237.0]	167.0 [124.0;226.0]	171.0 [126.2;213.8]	185.0 [130.8;259.2]	172.0 [128.5;247.8]	0.464
AG (mEq/L))	17.0 [14.0;20.0]	16.0 [14.0;19.0]	15.0 [12.0;18.0]	18.0 [15.0;21.0]	18.0 [15.0;21.0]	<0.001
ALT (IU/L)	55.0 [26.0;87.0]	56.0 [28.0;91.0]	45.5 [25.0;98.5]	49.5 [29.0;75.0]	57.0 [26.0;70.2]	0.752
Cr (mg/dL)	1.5 [1.0;2.0]	1.5 [1.1;2.0]	1.1 [0.9;1.5]	1.6 [1.2;2.5]	1.5 [1.2;2.1]	<0.001
PT (sec)	14.7 [13.1;17.2]	14.1 [12.9;15.5]	14.8 [13.1;17.2]	15.1 [13.5;19.4]	15.5 [13.4;18.2]	<0.001
APTT (sec)	42.4 [31.8;73.0]	44.5 [32.7;69.8]	39.0 [30.4;74.5]	42.3 [31.1;90.4]	38.1 [31.0;66.6]	0.481
pH (units)	7.3 [7.3;7.4]	7.4 [7.3;7.4]	7.3 [7.2;7.4]	7.3 [7.3;7.4]	7.3 [7.2;7.4]	<0.001
PaCO2 (mmHg)	40.0 [35.0;45.0]	40.0 [35.0;45.0]	41.0 [35.2;46.0]	39.0 [35.0;44.2]	40.0 [33.0;46.0]	0.738
PaO2 (mmHg)	87.0 [50.0;159.0]	81.0 [44.5;121.0]	126.0 [80.2;259.0]	86.0 [45.5;150.5]	97.5 [58.8;225.2]	<0.001
Lac (mmol/L))	2.2 [1.5;3.1]	2.0 [1.4;2.8]	2.2 [1.5;3.6]	2.2 [1.5;3.3]	3.0 [1.9;4.6]	<0.001
LDH (IU/L)	509.0 [363.0;735.0]	499.0 [359.0;728.0]	457.0 [318.8;629.0]	538.0 [395.0;929.8]	538.0 [367.2;663.5]	0.037
TnT (ng/mL)	1.3 [0.5;3.5]	1.3 [0.5;3.7]	1.4 [0.8;2.6]	1.8 [0.4;5.3]	1.0 [0.3;3.1]	0.409
Comorbidities						
Afib	261 (49.0%)	138 (45.0%)	33 (44.6%)	58 (60.4%)	32 (57.1%)	0.028
HTN	403 (75.6%)	233 (75.9%)	58 (78.4%)	68 (70.8%)	44 (78.6%)	0.62
DM	235 (44.1%)	134 (43.6%)	32 (43.2%)	50 (52.1%)	19 (33.9%)	0.181
HLP	292 (54.8%)	181 (59.0%)	43 (58.1%)	46 (47.9%)	22 (39.3%)	0.021
COPD	54 (10.1%)	37 (12.1%)	6 (8.1%)	5 (5.2%)	6 (10.7%)	0.245
PNA	119 (22.3%)	71 (23.1%)	12 (16.2%)	23 (24.0%)	13 (23.2%)	0.598
CKD	190 (35.6%)	111 (36.2%)	16 (21.6%)	42 (43.8%)	21 (37.5%)	0.027
Oral medications						
ACEI ARB	198 (37.1%)	134 (43.6%)	35 (47.3%)	18 (18.8%)	11 (19.6%)	<0.001
Beta Blocker	405 (76.0%)	235 (76.5%)	64 (86.5%)	71 (74.0%)	35 (62.5%)	0.016
Furosemide	478 (89.7%)	289 (94.1%)	68 (91.9%)	80 (83.3%)	41 (73.2%)	<.001
Spironolactone	45 (8.4%)	29 (9.4%)	9 (12.2%)	6 (6.2%)	1 (1.8%)	0.119
Vasoactive drugs						
Dobutamine	148 (27.8%)	85 (27.7%)	12 (16.2%)	38 (39.6%)	13 (23.2%)	0.007
Dopamine	149 (28.0%)	80 (26.1%)	18 (24.3%)	38 (39.6%)	13 (23.2%)	0.044
Epinephrine	137 (25.7%)	56 (18.2%)	31 (41.9%)	27 (28.1%)	23 (41.1%)	<.001
Norepinephrine	388 (72.8%)	195 (63.5%)	57 (77.0%)	86 (89.6%)	50 (89.3%)	<.001
Phenylephrine	189 (35.5%)	77 (25.1%)	33 (44.6%)	46 (47.9%)	33 (58.9%)	<.001
Other indicators						
Ventilation	508 (95.3%)	289 (94.1%)	73 (98.6%)	92 (95.8%)	54 (96.4%)	0.456
CRRT	99 (18.6%)	24 (7.8%)	5 (6.8%)	49 (51.0%)	21 (37.5%)	<.001
Sepsis	441 (82.7%)	240 (78.2%)	57 (77.0%)	90 (93.8%)	54 (96.4%)	<.001
AKI	859 (98.1%)	128 (96.2%)	293 (98.3%)	308 (98.4%)	130 (98.5%)	0.478
SOFA	8.0 [5.0;11.0]	9.0 [6.0;11.0]	7.0 [4.0;9.0]	8.0 [6.0;11.0]	10.5 [8.8;13.0]	<0.001
Charlson	7.0 [5.0;9.0]	7.0 [5.0;9.0]	6.0 [5.0;8.0]	8.0 [6.0;9.0]	7.0 [5.0;9.0]	0.007
Hosp Survival Day	412 [13, 4442]	1055 [18, 4442]	3001 [101, 4442]	20 [7, 1170]	14 [7, 2901]	<0.001
Is Dead	322 (60.4%)	167 (54.4%)	39 (52.7%)	74 (77.1%)	42 (75.0%)	<0.001
30-day mortality	199 (37.3%)	97 (31.6%)	12 (16.2%)	57 (59.4%)	33 (58.9)	<0.001

T, Temperature; HR, heart Rate; RR, respiratory rate; SBP, systolic blood pressure; SpO₂, peripheral capillary Oxygen saturation; WBC, white blood cell; PLT, Platelet; Hb, hemoglobin; RDW, red cell distribution width; Na, sodium; K, potassium; Ca, calcium; GLU, glucose; AG, anion gap; ALT, alanine aminotransferase; Cr, creatinine; PT, prothrombin Time; APTT, activated partial thromboplastin time; pH, hydrogen ion concentration; PaCO2, partial pressure of carbon dioxide; PaO₂, partial pressure of oxygen; Lac, lactate; LDH, lactate dehydrogenase; TnT, troponin T; Afib, atrial fibrillation; HTN, hypertension; DM, diabetes mellitus; HLP, hyperlipidemia; COPD, chronic obstructive pulmonary disease; PNA, pneumonia; CKD, chronic kidney disease; ACEI ARB, angiotensin-converting enzyme inhibitor/angiotensin II receptor blocker; Beta Blocker, beta-adrenergic blocking agent; CRRT, continuous renal replacement therapy; AKI, acute kidney injury; SOFA, sequential organ failure assessment; Charlson, charlson comorbidity index.

### 3.2 FB trajectory description

Prior to trajectory grouping for FB, we pre-checked the FB from day 1 to day 7. However, due to substantial missing values, with missing rates from day 4 to day 7 being 5.7%, 23.9%, 39.3%, and 50.6%, respectively, we only included FB data for days 1–4. Table 2 shows the model fitting statistics used to determine the optimal number of FB trajectory groups. The 4-group model had an AIC value of 19,937.75, which was close to that of the 5-group model (19,836.05). Both models met the AvePP and minimum OCC criteria. However, in the 5-group model, some subgroups accounted for only 9.2% of the total population, resulting in an insufficient sample size for stable interpretation. Therefore, considering model interpretability and simplicity, the 4-group trajectory model was selected as the optimal model.

**Table 2:** Performance of the group-based trajectory model for fluid balance trajectories.

Trajectories	BIC	AIC	AvePP	Minimum OCC	Class proportion
1 group	20502.40	20479.74	1.00	NaN	100.0%
2 groups	20143.55	20092.56	0.88/0.90	5.48	61.7%/38.3%
3 groups	20110.91	20025.94	0.93/0.88/0.80	6.52	69.0%/17.4%/13.6%
4 groups	20056.71	19937.75	0.91/0.81/0.81/0.90	7.90	58.3%/13.7%/17.7%/10.4%
5 groups	19989.00	19836.05	0.85/0.84/0.81/0.81/0.91	8.41	44.6%/9.2%/14.8%/21.1%/10.3%

BIC, Bayesian information criterion, AIC, Akaike information criterion, AvePP, average posterior probability, OCC, odds of correct classification.

**Figure 2:**
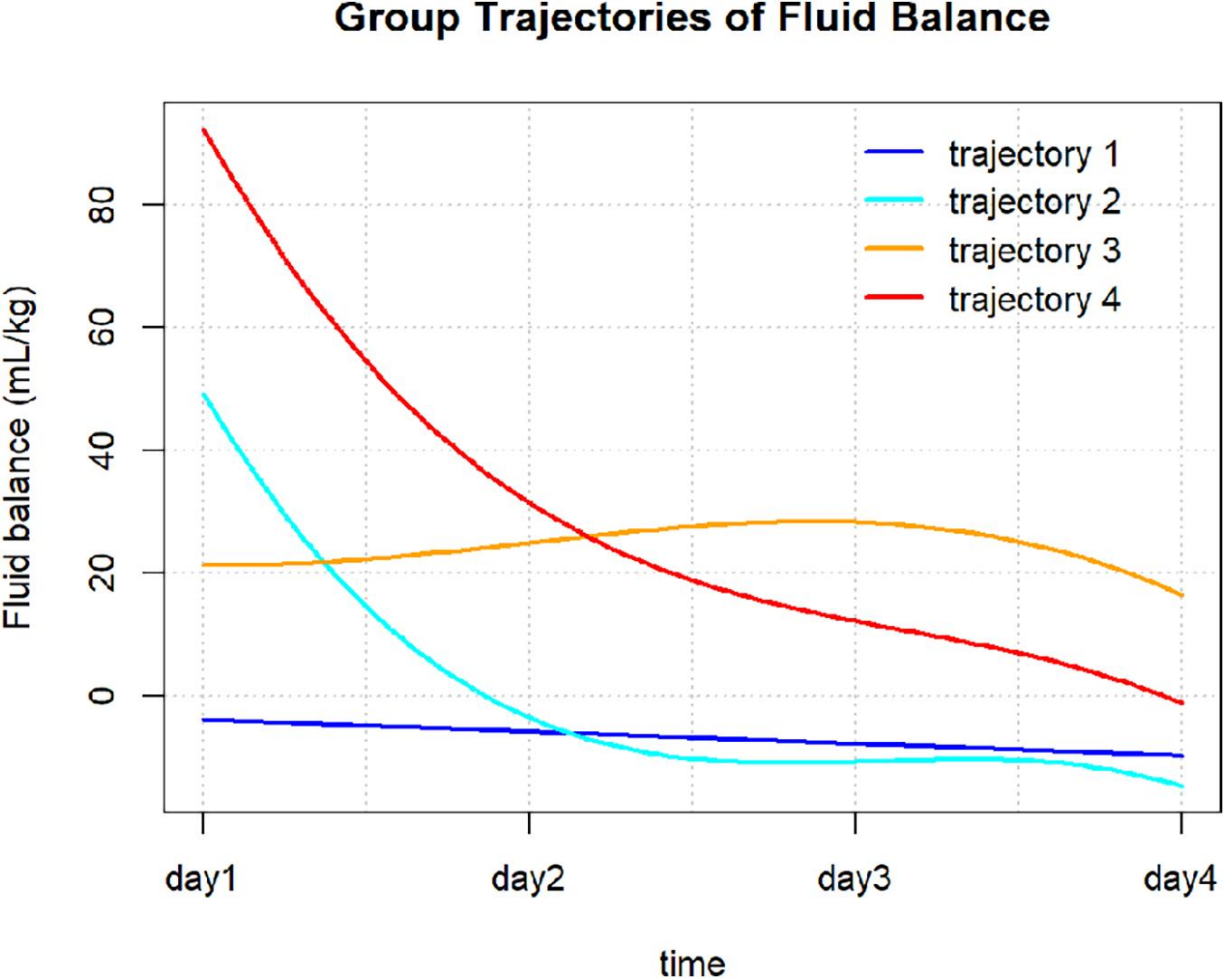
Fluid balance trajectories in patients with AMI-CS.

The FB trajectory groups are shown in Figure 2. Trajectory 1 (stable negative balance) included 307 (57.6%) patients, with FB consistently below 0 mL/kg. Trajectory 2 (rapid decline to negative balance) included 74 (13.9%) patients, with FB around 50 mL/kg on day 1, rapidly declining to 0 mL/kg by day 2, and maintaining negative balance on days 3 and 4. Trajectory 3 (persistent positive balance) included 96 (18.0%) patients, with FB fluctuating around 0 mL/kg, but always remaining in positive balance. Trajectory 4 (high-level decreasing) included 56 (10.5%) patients, with FB highest on day 1, greater than 80 mL/kg, followed by a rapid decrease, but remaining in positive balance during the first four days. Significant differences in baseline characteristics were observed between the different FB trajectory groups (as shown in Table 1). Patients in group 4 were younger, with a higher proportion of females, and a greater proportion of those with CRRT, sepsis, and CKD, along with the highest SOFA scores. Group 3 had higher usage rates of dobutamine and norepinephrine. Group 2 patients were younger, had higher body weight, and lower heart rate and SOFA scores. In contrast, group 1 had intermediate overall indicators but a higher proportion of ACEI/ARB and diuretic use.

### 3.3 Association of FB trajectories, FO Status, and survival

**Figure 3:**
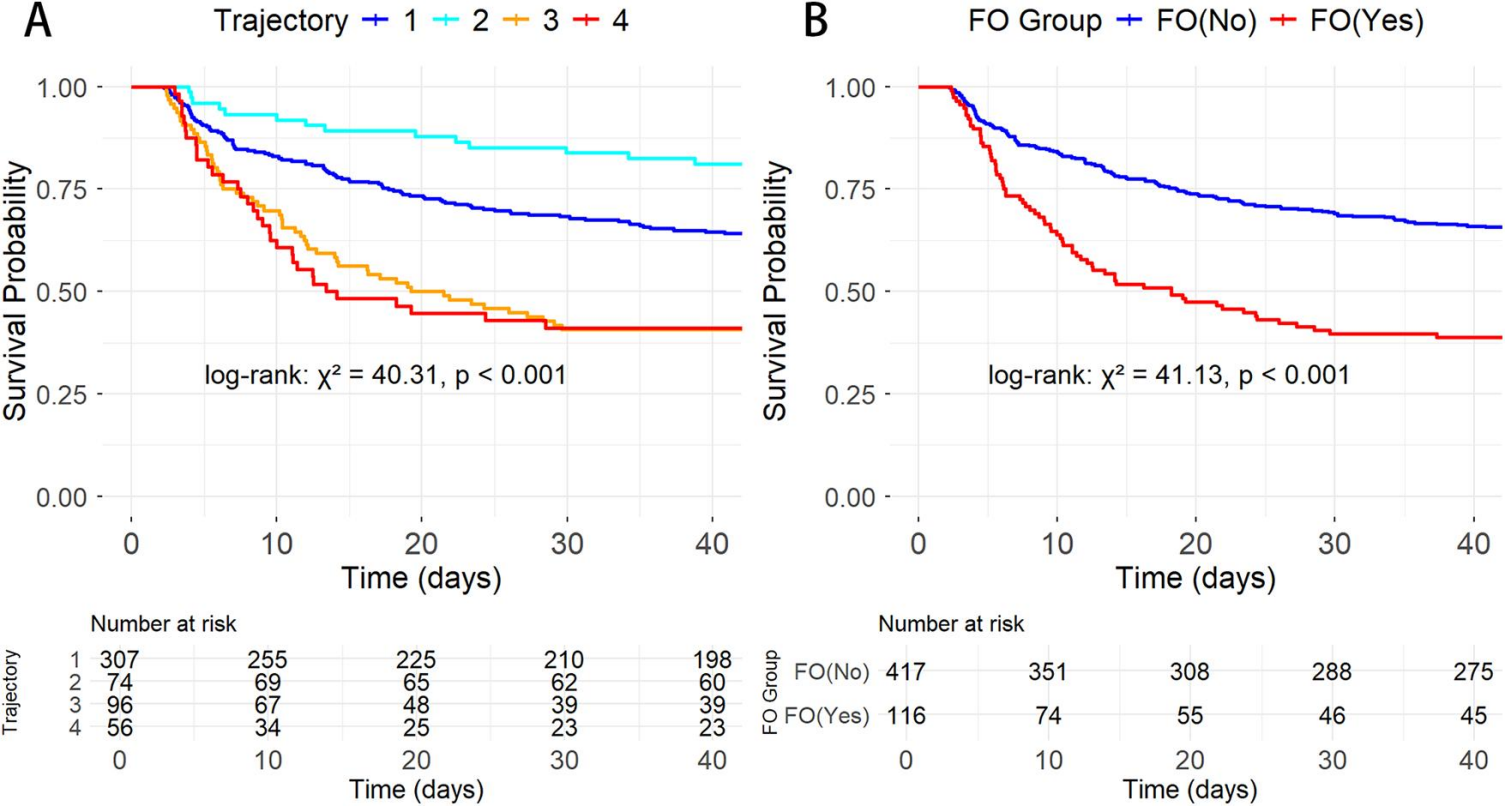
K–M survival curves showing the relationship between different AMI-CS groups and 30-day mortality. **(A)** By FB trajectory groups; **(B)** By FO status.

Kaplan–Meier survival analysis revealed significant survival differences between FB trajectory groups and FO status (Figure 3). There were significant differences in survival rates between the trajectory groups (log-rank:*χ*^2^ = 40.31, *P* < 0.001), with trajectories 1 (stable negative balance) and 2 (rapid decline to negative balance) showing better survival compared to trajectories 3 (persistent positive balance) and 4 (high-level decreasing). Additionally, the mortality risk in the FO group was significantly higher than in the non-overload group (log-rank: *χ*^2^ = 41.13, *P* < 0.001).

The univariate and multivariate Cox regression results are shown in Supplementary Table S1. The results indicate that trajectory groups, age, weight, Na, AG, ACEI/ARB, and β-blockers were associated with AMI-CS prognosis. Four Cox regression models were constructed based on different covariates (Table 3). After adjusting for age, weight, sodium (Na), anion gap (AG), and medication use (ACEI/ARB, β-blockers), trajectory 3 (HR = 1.78, 95% CI: 1.19–2.66, *P* = 0.005) significantly increased the risk of adverse outcomes, while trajectory 4 (HR = 1.56, 95% CI: 0.99–2.46, *P* = 0.053) approached significance. Trajectory 1 (HR = 0.99, 95% CI: 0.70–1.41, *P* = 0.971) showed no difference compared to the reference group, trajectory 2.

**Table 3:** Cox regression multimodel analysis of fluid balance trajectories in AMI-CS patients.

Variables	Model 1		Model 2		Model 3		Model 4	
	HR (95%CI)	*P*	HR (95%CI)	*P*	HR (95%CI)	*P*	HR (95%CI)	*P*
Trajectory								
2	1.00 (Reference)		1.00 (Reference)		1.00 (Reference)		1.00 (Reference)	
1	1.19 (0.84∼1.68)	0.338	1.16 (0.82∼1.64)	0.406	1.09 (0.77∼1.56)	0.618	0.99 (0.70∼1.41)	0.971
3	2.40 (1.63∼3.54)	<.001	2.61 (1.76∼3.85)	<.001	2.28 (1.53∼3.40)	<.001	1.78 (1.19∼2.66)	0.005
4	2.26 (1.46∼3.50)	<.001	2.18 (1.40∼3.39)	<.001	1.89 (1.20∼2.96)	<.001	1.56 (0.99∼2.46)	0.053

HR, hazard ratio; CI, confidence interval.

Model 1: Crude.

Model 2: Adjust: Age, Weight.

Model 3: Adjust: Age, Weight, Na, AG.

Model 4: Adjust: Age, Weight, Na, AG, ACEI.ARB, beta.blocker.

### 3.4 Subgroup analysis

**Figure 4:**
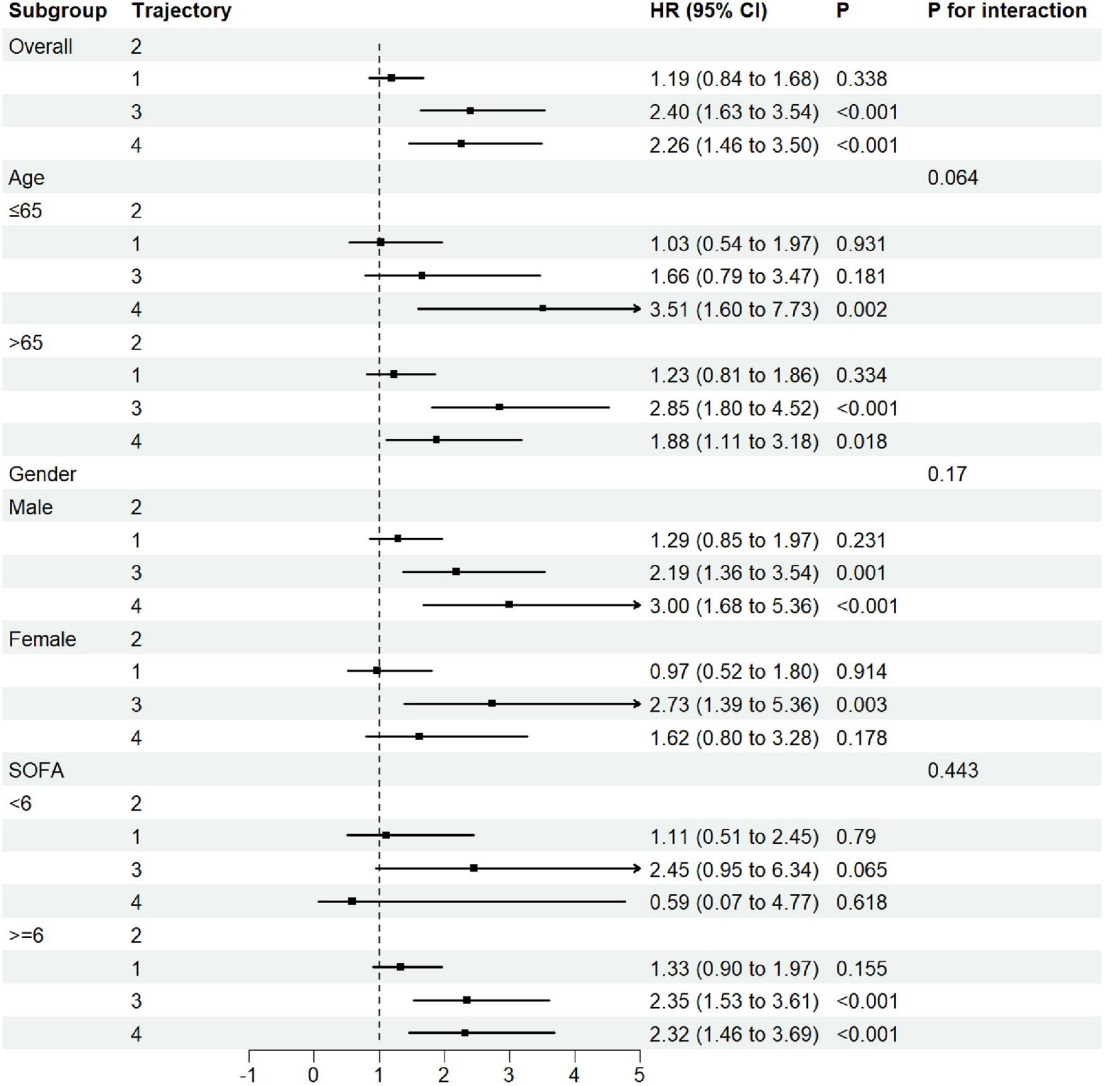
Forest subgroup analysis FB trajectories and 30-Day ICU mortality in AMI-CS patients.

The forest plot for subgroup analysis (Figure 4) demonstrated that compared to trajectory 2, trajectories 3 and 4 consistently increased the risk of major adverse events in AMI-CS patients. This trend was consistent across all predefined subgroups, with no statistically significant interaction (*P* for interaction >0.05). While differences in age, gender, or SOFA score did not significantly alter the overall association, trajectory 4 was particularly prominent in patients aged ≤65 years (HR 3.51, 95% CI 1.60–7.73, *P* = 0.002) and in male patients, where the HR exceeded 3.00 (95% CI 1.68–5.36, *P* < 0.001). In addition, subgroup analyses stratified by sepsis and norepinephrine use (Supplementary Figure S4) showed trends consistent with the main findings. Among patients with sepsis or those receiving norepinephrine therapy, trajectories 3 and 4 remained consistently associated with a higher risk of mortality.

### 3.5 Sensitivity analysis

Sensitivity analysis further validated the robust association between FB trajectories and prognosis in AMI-CS patients. By excluding patients with low blood pressure (<90 mmHg) at ICU admission (remaining 460 patients) and those with CKD (remaining 343 patients), the potential confounding effects of hypotension and renal dysfunction on FB were minimized. The FB trajectory remained consistent with the primary analysis, showing similar potential changes (Supplementary Figures S5 and S6). A key finding is that the 30-day all-cause mortality rate in the rapid transition to negative balance group and the stable negative balance group remained significantly lower than in the other groups, which was completely consistent with the baseline analysis.

## 4 Discussion

This study employed the GBTM method to explore the FB trends in ICU patients with AMI-CS and identified a correlation between FB trajectory levels and mortality risk in these patients. We identified four FB trajectories: Trajectory 1 (stable negative balance), Trajectory 2 (rapid decline to negative balance), Trajectory 3 (persistent positive balance), and Trajectory 4 (high-level decreasing). After adjusting for all confounding factors, we observed that the 30-day mortality risk was lower in the stable negative balance or rapid decline to negative balance groups. Subgroup and sensitivity analyses yielded similar results. Additionally, we also observed that FO was associated with an increased mortality risk.

The clinical course of cardiogenic shock after acute myocardial infarction (AMI) follows a dynamic evolutionary spectrum, and accurately assessing the in-hospital mortality of AMI-CS patients is crucial for the rational allocation of limited medical resources, optimizing clinical decisions, and reducing healthcare costs (21). Several scoring systems have been developed to assess the prognosis of AMI-CS patients (22–25), and prognostic factors vary, including age, smoking, hypertension, diabetes, stroke history, heart rate (HR), lactate levels, pH, coagulation function, urea nitrogen, NT-proBNP, and others. Fluid therapy plays a critical role in maintaining tissue perfusion in the management of cardiogenic shock. During the early stages of treatment, most AMI-CS patients receive fluid therapy (7) and vasopressor treatment to improve low perfusion and/or hypotension (26). However, the rationale for fluid administration in AMI-CS should be carefully considered, as the underlying pathophysiology often involves severe ventricular dysfunction and elevated filling pressures. In such cases, cardiac output typically fails to increase in response to volume loading, whereas pulmonary and systemic congestion may worsen (5, 7, 27). Current guidelines and evidence suggest that fluid resuscitation should be considered first-line treatment for ACS-related CS patients unless signs of FO are present (5). However, fluid therapy remains controversial in the treatment of AMI-CS patients, requiring a balance between achieving adequate volume resuscitation and avoiding fluid overload.

In recent years, the side effects of fluid resuscitation—positive FB and FO—have become a growing concern (28). Our study found a significant association between different FB trajectories and 30-day mortality in AMI-CS patients. Among them, the risk of mortality was lower in the negative fluid balance or rapid negative fluid balance groups compared to the persistent positive balance group. After adjusting for hypotension and CKD at ICU admission, the consistent conclusion was observed. Furthermore, the mortality risk in the FO group was significantly higher than in the non-overload group. Similarly, some studies have found that early positive FB or FO in patients with cardiogenic shock is significantly associated with higher mortality (12, 13). Moreover, in STEMI patients with cardiogenic shock, positive FB was significantly associated with higher-stage acute kidney injury (AKI) and lower AKI recovery rates (14). Some studies have reported that, in patients admitted to the ICU with severe heart failure and/or cardiogenic shock, fluid overload at ICU discharge was not significantly associated with 30-day mortality (15). However, it is important to note that this study focused on fluid load status at discharge, which is inconsistent with other studies' observation time points and the definition of FO.

The pathophysiological mechanisms further explain the association between positive FB or FO and poor prognosis in AMI-CS patients. Myocardial ischemic injury leads to ventricular dysfunction and systemic hypoperfusion, which in turn activates the sympathetic nervous system and the renin-angiotensin-aldosterone system, increasing preload and circulating plasma volume. This pathophysiological mechanism is particularly evident in cardiogenic shock (CS) patients, and if the congestive state is not rapidly relieved, it may lead to a vicious cycle, further exacerbating myocardial ischemia and circulatory failure (1). The core goal of fluid resuscitation is to restore hemodynamic stability through volume expansion (27), but excessive positive fluid load may worsen cardiac preload, especially in cases of severe cardiac dysfunction. The Frank-Starling mechanism suggests that excessive filling of the heart may reduce myocardial contractility, subsequently decreasing stroke volume and cardiac output (29). Furthermore, in patients with right ventricular dysfunction, FO may lead to right ventricular dilation, further impairing left ventricular filling and exacerbating systemic hypoperfusion (30). Therefore, the effect of fluid therapy depends on the patient's fluid responsiveness and cardiac function status, and excessive fluid use may lead to poor prognosis, highlighting the need for individualized fluid therapy. Interestingly, the rapid transition from an early positive to a negative fluid balance (trajectory 2) was associated with better outcomes. This pattern may reflect an appropriate fluid management strategy in AMI-CS, where initial fluid administration restores perfusion during the acute phase, followed by timely de-resuscitation to prevent fluid overload and congestion.

This study is the first to systematically evaluate the relationship between FB trajectories and prognosis in ICU AMI-CS patients. Using the GBTM method, we classified the dynamic FB patterns in AMI-CS patients and identified high-risk FB trajectory groups. This could aid in clinical practice by enabling targeted care and early intervention for high-risk patient populations. However, there are certain limitations in this study. First, it is based on a single-center database, which introduces selection bias. Additionally, as a retrospective observational study, the relationship between FO trajectory grouping and ICU mortality can only be inferred, and causality needs to be established through further prospective studies. Second, baseline characteristics were not fully adjusted between the different trajectory groups, and baseline characteristics may influence prognosis in different trajectory groups. Our primary goal was to classify the fluid changes in AMI-CS patients, and we further assessed the stability of these results through covariate adjustment, subgroup analysis, and sensitivity analysis. Third, we excluded patients who died within 72 h of admission because such patients could not be further described regarding fluid dynamic trajectories. AMI-CS patients have a high mortality rate, and excluding those who died early may underestimate the impact of trajectory on mortality. In addition, some key clinical parameters such as SCAI classification, LVEF, coronary anatomy and revascularization data, use of mechanical circulatory support, and cardiac arrest status were unavailable or incomplete in the MIMIC-IV database. The exclusion of these variables may partially explain the observed differences among trajectory groups and represents an important limitation of this study. Finally, despite adjusting for confounders as much as possible, there may still be unadjusted confounding factors, such as unrecorded diuretic doses or echocardiography results, which may affect the accuracy and completeness of the study.

## 5 Conclusion

This study explored the relationship between FB trajectories and 30-day mortality in ICU AMI-CS patients. Using the GBTM method, we identified four different FB change trajectories, with persistent positive balance associated with a higher risk of mortality. Fluid overload increased the risk of death in AMI-CS patients. Fluid therapy should be based on pathophysiological considerations and underlying causes, and after evaluating fluid responsiveness, it should be implemented accordingly. Monitoring FB trajectories may help identify high-risk AMI-CS individuals for targeted care and treatment.

## Data Availability

The original contributions presented in the study are included in the article/Supplementary Material, further inquiries can be directed to the corresponding author.
