# Supplementary figures and images for: Fluid balance trajectories and prognosis in patients with acute myocardial infarction complicated by cardiogenic shock: a group-based trajectory model approach
Source: Front Cardiovasc Med. 2026 Jan 5;12:1674197. doi: 10.3389/fcvm.2025.1674197 (PMC12812695; doi:10.3389/fcvm.2025.1674197)

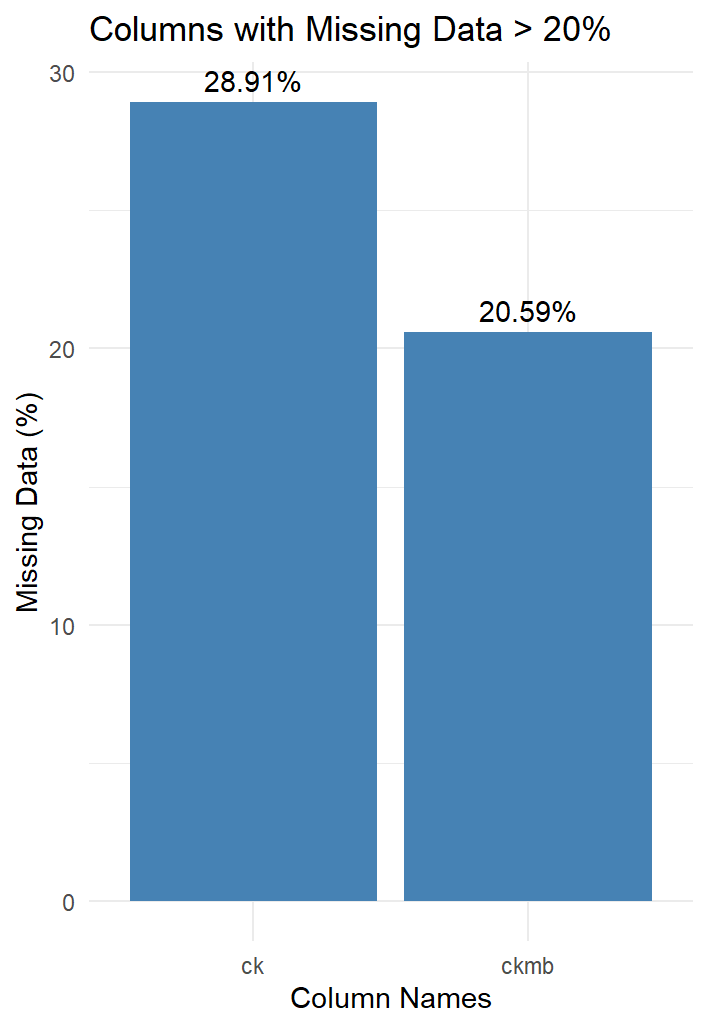

Supplement: Supplementary file 2 [file Image1.tiff]

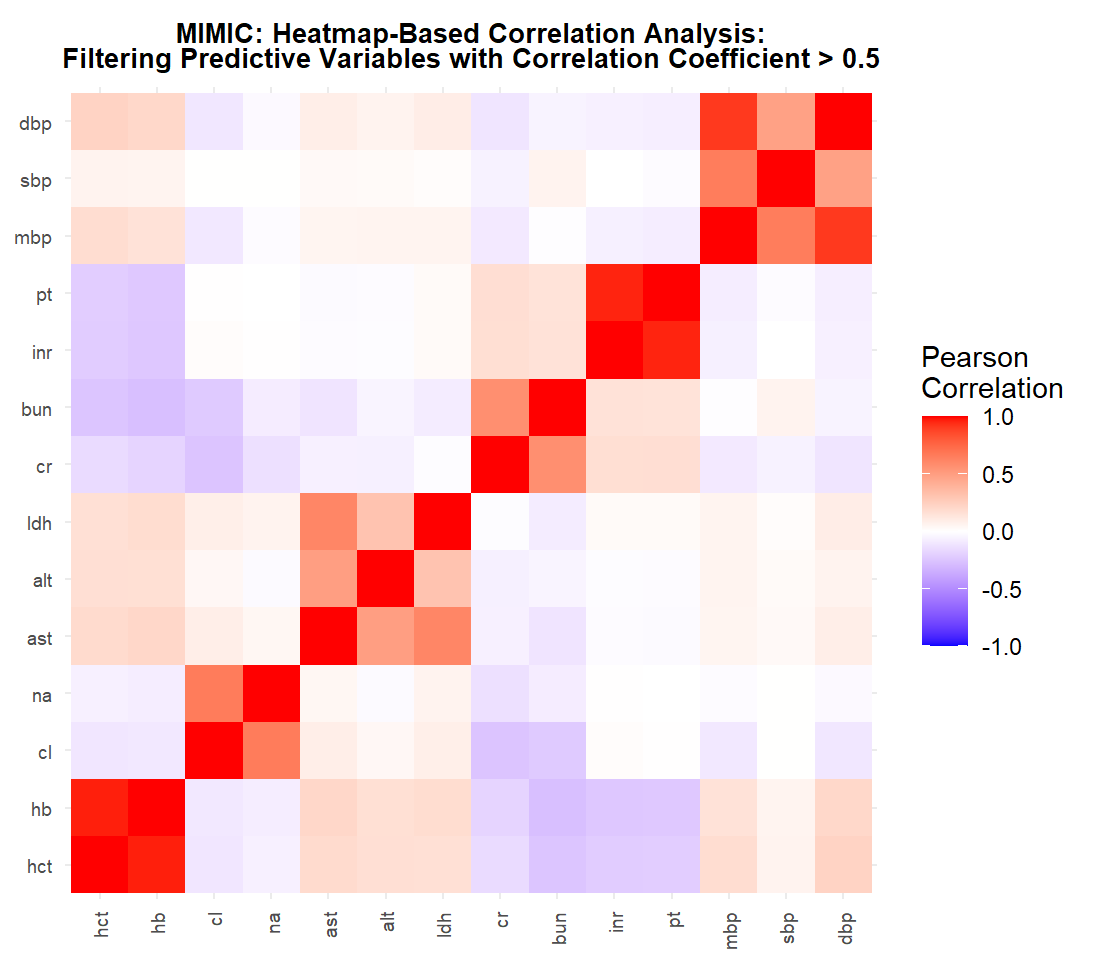

Supplement: Supplementary file 3 [file Image2.tiff]

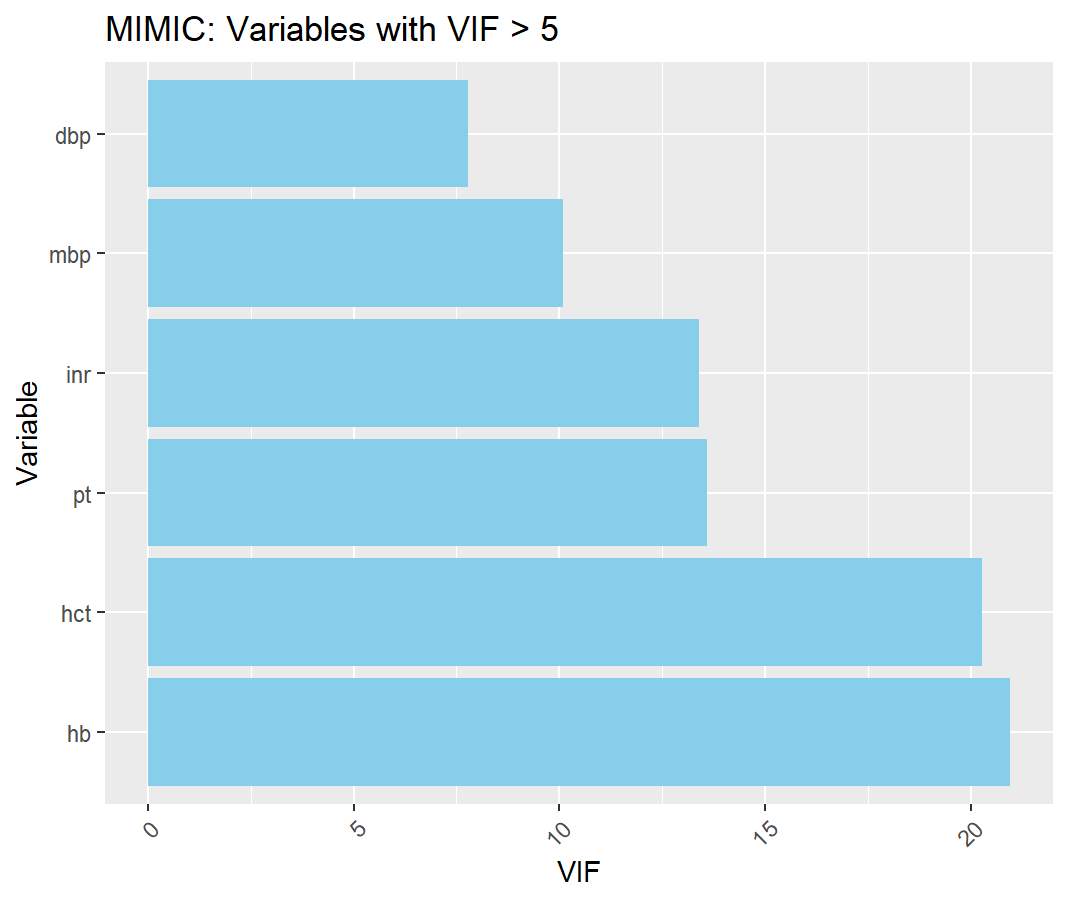

Supplement: Supplementary file 4 [file Image3.tiff]

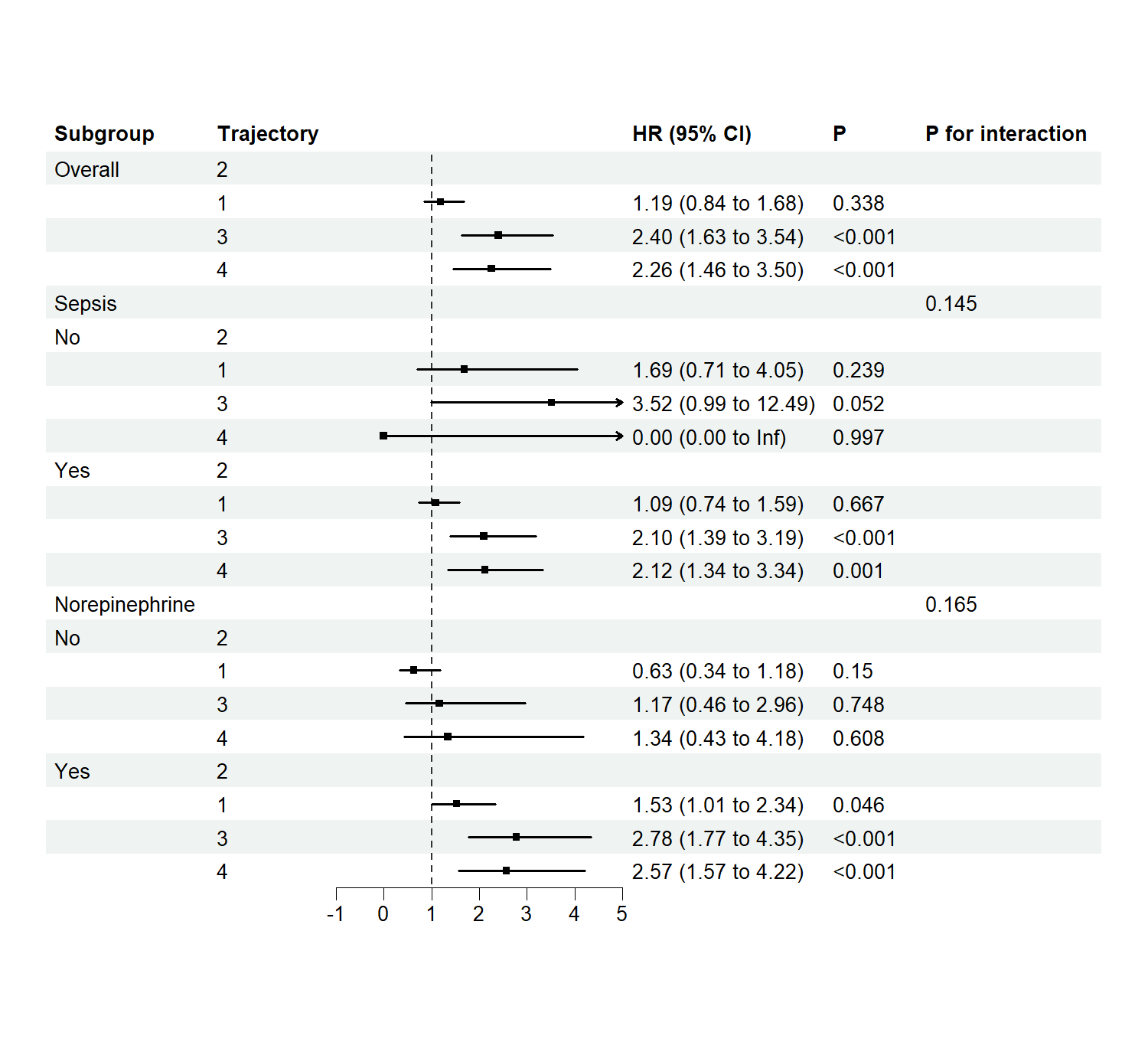

Supplement: Supplementary file 5 [file Image4.tiff]

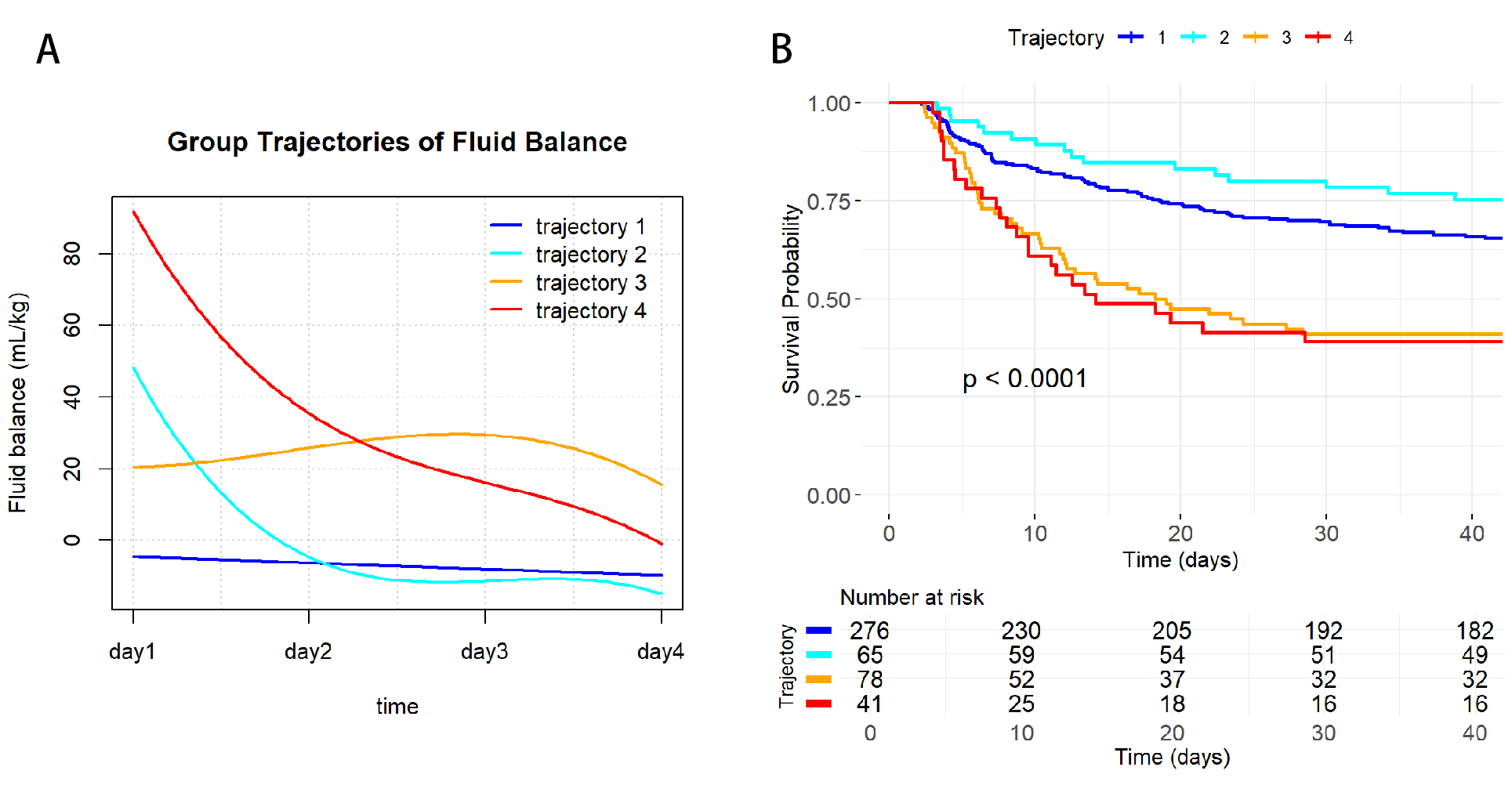

Supplement: Supplementary file 6 [file Image5.tiff]

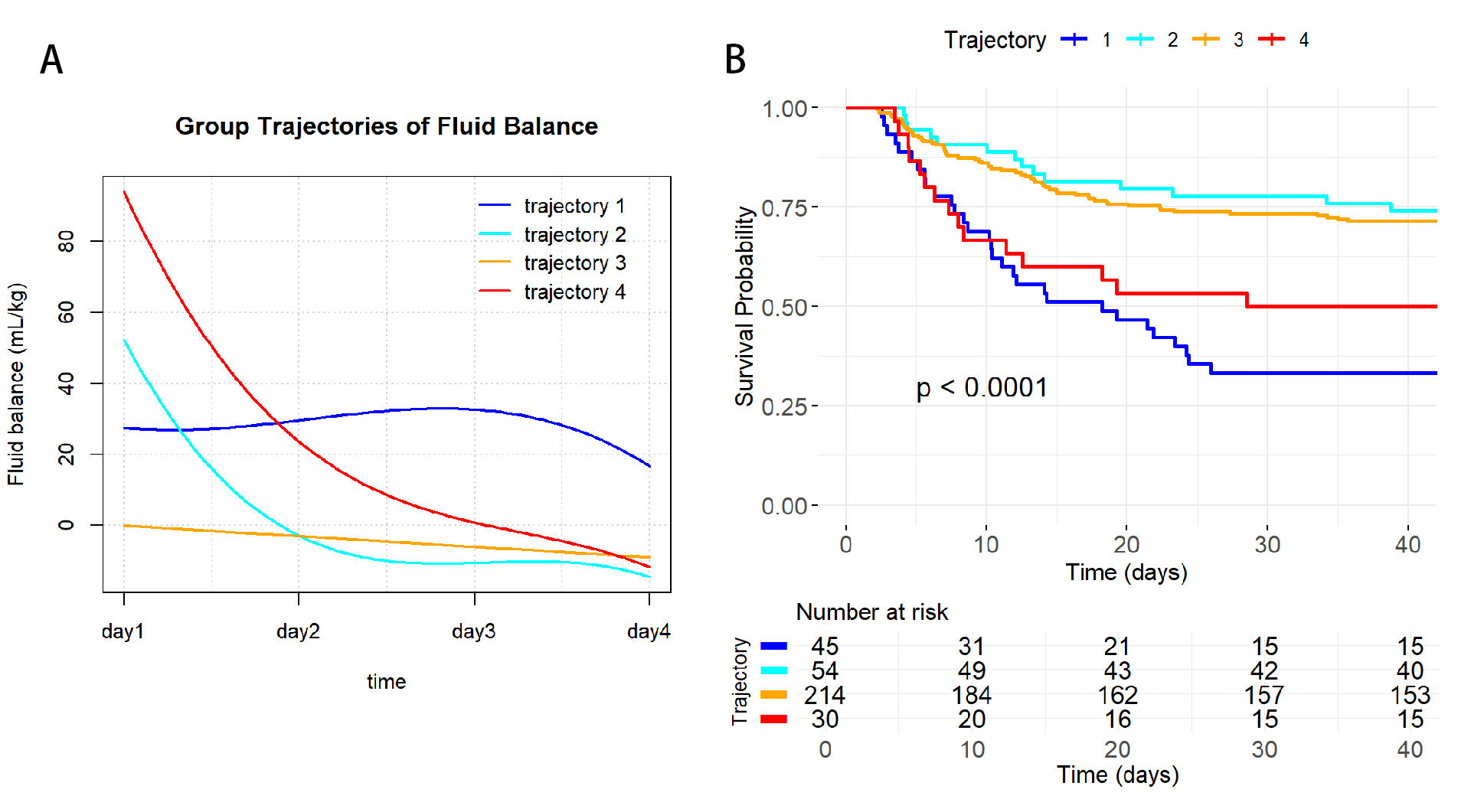

Supplement: Supplementary file 7 [file Image6.tiff]
